# Supplementary material for: An Improved Isotope Labelling Method for Quantifying Deamidated Cobratide Using High-Resolution Quadrupole-Orbitrap Mass Spectrometry
Source: Molecules. 2022 Sep 20;27(19):6154. doi: 10.3390/molecules27196154 (PMC9572859; doi:10.3390/molecules27196154)
Supplement: Supplementary file 1 [file molecules-27-06154-s001.zip › molecules-1886380-supplementary.pdf]

# An improved isotope labelling method for quantification of deamidated cobratide using high-resolution quadrupole-Orbitrap mass spectrometry

**Bo Liu[1,2,#], Lu Huang[1,2,#], Rongrong Xu[1,2], Huihong Fan[1,2,*] and Yue Wang[1,2]**

1 National Institutes for Food and Drug Control, 31st Huatuo Rd., Daxing Dist., Beijing, 102629, China

2 NMPA Key Laboratory for Quality Research and Evaluation of Chemical Drugs, Beijing 102629, China

# These authors contributed equally to this work.

* Correspondence: shenghuayaoshi@126.com

**Table S1.** The isotopic peaks in extracted ion chromatograms (EICs) for Asn, Asp and iso-Asp

| Peak | m/z | Extracted mass range |
|---|---|---|
| Asn | 479.226 | 479.22414-479.22703 |
| | 479.559 | 479.55798-479.56086 |
| | 479.893 | 479.89144-479.89432 |
| | 480.227 | 480.22518-480.22806 |
| | 480.560 | 480.55868-480.56156 |
| | 480.894 | 480.89254-480.89542 |
| | 481.228 | 481.22631-481.22919 |
| Asp and iso-Asp | 479.554 | 479.55294-479.55582 |
| | 479.888 | 479.88708-479.88996 |
| | 480.222 | 480.22030-480.22318 |
| | 480.555 | 480.55410-480.55698 |
| | 480.889 | 480.88754-480.89042 |
| | 481.223 | 481.22137-481.22425 |
| | 481.556 | 481.55517-481.55805 |
| | 481.890 | 481.88911-481.89201 |
